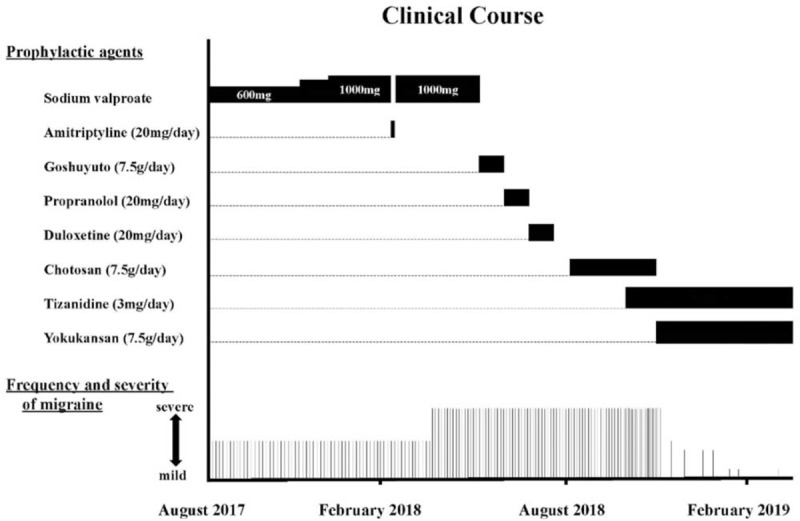# Effectiveness of the traditional Japanese Kampo medicine Yokukansan for chronic migraine: A case report: Erratum

**DOI:** 10.1097/MD.0000000000017380

**Published:** 2019-09-27

In the article, “Effectiveness of the traditional Japanese Kampo medicine Yokukansan for chronic migraine: A case report”,^[1]^ which appears in Volume 98, Issue 36 of *Medicine*, four of the boxes in Figure 2 appeared in white instead of black during publication. The corrected figure appears below.

**Figure:**